# Heat Transfer Enhancement in Unsteady MHD Natural Convective Flow of CNTs Oldroyd-B Nanofluid under Ramped Wall Velocity and Ramped Wall Temperature

**DOI:** 10.3390/e22040401

**Published:** 2020-03-31

**Authors:** Talha Anwar, Poom Kumam, Ilyas Khan, Wiboonsak Watthayu

**Affiliations:** 1 Department of Mathematics, Faculty of Science, King Mongkut’s University of Technology Thonburi (KMUTT), 126 Pracha-Uthit Road, Bang Mod, Thrung Khru, Bangkok 10140, Thailand; anwartalha80@gmail.com (T.A.); wiboonsak.wat@kmutt.ac.th (W.W.); 2 KMUTT Fixed Point Research Laboratory, Room SCL 802 Fixed Point Laboratory, Science Laboratory Building, Department of Mathematics, Faculty of Science, King Mongkut’s University of Technology Thonburi (KMUTT), Bangkok 10140, Thailand; 3 Center of Excellence in Theoretical and Computational Science (TaCS-CoE), Science Laboratory Building, Faculty of Science, King Mongkut’s University of Technology Thonburi (KMUTT), 126 Pracha-Uthit Road, Bang Mod, Thrung Khru, Bangkok 10140, Thailand; 4 Department of Medical Research, China Medical University Hospital, China Medical University, Taichung 40402, Taiwan; 5 Department of Mathematics, College of Science Al-Zulfi, Majmaah University, Al-Majmaah 11952, Saudi Arabia; i.said@mu.edu.sa

**Keywords:** carbon nanotubes, Laplace transform, ramped wall, MHD, heat transfer, Oldroyd-B nanofluid

## Abstract

This article analyzes heat transfer enhancement in incompressible time dependent magnetohydrodynamic (MHD) convective flow of Oldroyd-B nanofluid with carbon nanotubes (CNTs). Single wall carbon nanotubes (SWCNTs) and multi-wall carbon nanotubes (MWCNTs) are immersed in a base fluid named Sodium alginate. The flow is restricted to an infinite vertical plate saturated in a porous material incorporating the generalized Darcy’s law and heat suction/injection. The governing equations for momentum, shear stress and energy are modelled in the form of partial differential equations along with ramped wall temperature and ramped wall velocity boundary conditions. Laplace transformation is applied to convert principal partial differential equations to ordinary differential equations first and, later, complex multivalued functions of Laplace parameter are handled with numerical inversion to obtain the solutions in real time domain. Expression for Nusselt number is also obtained to clearly examine the difference in rate of heat transfer. A comparison for isothermal wall condition and ramped wall condition is also made to analyze the difference in both profiles. A graphical study is conducted to analyze how the fluid profiles are significantly affected by several pertinent parameters. Rate of heat transfer increases with increasing volume fraction of nanoparticle while shear stress reduces with elevation in retardation time. Moreover, flow gets accelerated with increase in Grashof number and Porosity parameter. For every parameter, a comparison between solutions of SWCNTs and MWCNTs is also presented.

## 1. Introduction

In emerging and modern technologies, non-Newtonian fluids are acquiring attention because of their higher practical significance. Examples of non-Newtonian fluids are honey, paints, toothpaste, polymer solutions and greases. In order to predict the features of such fluids, there exist many models. The relation which links shear rate and shear stress is nonlinear for non-Newtonian fluids. Therefore, the resulting flow equations associated to non-Newtonian fluids have higher order and are more complex than Navier Stokes equation. Due to these additional non linear terms, such fluids are hard to tackle. The purpose of forecasting the flow profile of non-Newtonian fluids together with handling non linear terms effectively is served by producing several mathematical models. The three principal types of such models are named integral, rate and differential models. Integral models incorporate substances like polymers which melt with noticeable memory. In such models, deformation gradient provides information about stress. While, there exists an implicit relation between stress and its higher order derivatives in rate type fluids. On the other hand, stress for fluids lying in the category of differential model is derived by its several higher derivatives.

In the current work, a subdivision of rate type fluid named Oldroyd-B fluid is selected due to its relatively higher significance, when it comes to prediction of both memory and elastic effects [1]. This model even preserves rheological effects for flows in one direction and for extensional flows it has non-physical singularity. This model was first given by James G. Oldroyd to anticipate the viscous and elastic profile of fluids. This model can be viewed as a generalization of Upper Convected Maxwell model, when viscosity of solvent is zero. Moreover, Maxwell material and viscous fluid are special cases of this model [2,3]. Das et al. studied the impact of magnetic field on oldroyd-B nanofluid for porous surface [4]. Subbarao et al. investigated the behavior of Oldroyd-B nanofluid under thermal radiation for stretching sheet [5]. Gupta et al. obtained the numerical solutions for three dimensional flow of Oldroyd-B nanofluid for bidirectional moving sheet [6]. The effect of thermophoresis on Oldroyd-B nanofluid flow was examined by Awad et al. [7]. Khan et al. provided the variation in oldroyd-B nanofluid, when heat is consumed or generated [8].

All aforementioned studies of Oldroy-B fluid do not involve the tube-shaped nanoparticles named carbon nanotubes (CNTs). These CNTs can be bent without any harm and have greater thermal conductivity and mechanical strength, when compared with other types of nanoparticles. These properties make them appealing and more applicable for practical purposes. In present study, two types of CNTs named SWCNTs and MWCNTs are considered. CNTs are said to be best heating conductors. CNTs applications include advanced electrodes, energy storage, conductive films and coatings (including transparent conductive coatings), solar, wearable electronics, thermal interface materials, structural materials, catalyst supports, biomedical and sensor applications.

**Figure 1:**
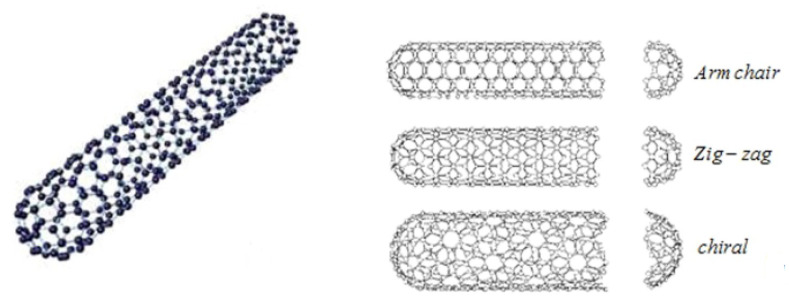
(**a**) Structure of single wall carbon nanotubes (SWCNTs). (**b**) Structure of three types of carbon nanotubes (CNTs).

**Figure 2:**
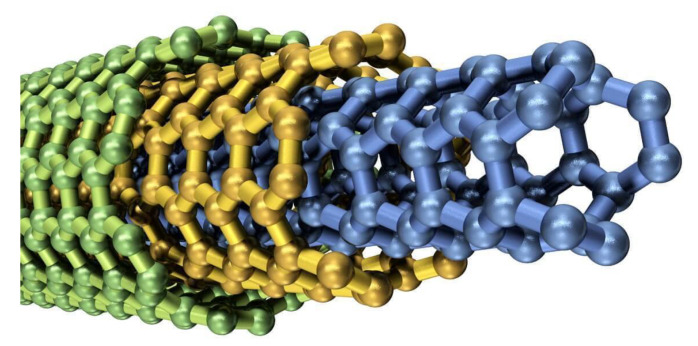
Structure of multi-wall carbon nanotubes (MWCNTs).

SWCNTs are defined as one dimensional, cylindrical shaped allotropes of carbon that have a high surface area and aspect ratio as shown in Figure 1a [9]. In Figure 1b [9], three different kinds of structures of SWCNTs (Armchair, Zigzag, Chiral) are presented. MWCNTs consist of multiple rolled layers (concentric tubes) of graphene layers in one dimensional format. The properties of MWCNTs are unique because they come in a complex array of forms and each concentric nanotube can have a different structure as shown in Figure 2 [9]. MWCNTs enhance the thermal, electrical and mechanical strength of the connected material, therefore they are point of interest for researchers these days.

The theme of nanofluids was initiated by Choi [10], when he suspended nano-sized solid particles in base fluid and the successive fluid was called nanofluid. Nanofluids have different sizes, types, and shapes depending upon the suspended nanoparticles, see [11,12,13,14,15,16]. Eid et al. employed finite element method to evaluate the solution of Blood-based SWCNTs flow through a circular cylinder in presence of electromagnetic radiation and a porous medium [17]. Boumaiza et al. studied analytical and numerical solutions for mixed convection Falkner-skan flow of nanofluids with variable thermal conductivity [18]. Effects of inclined magnetic field and variable thermal conductivity on heat transfer of squeezing unsteady nanofluid flow were analyzed by Lahmar et al. [19]. Eid et al. investigated the effects of convective condition and nanoparticles’ shapes on flow of non-Newtonian bio-nanofluids in blowing/suction process [20]. Combination of ramped boundary conditions and nanofluid is of effective significance physically but there is a dearth of articles in literature incorporating the solution of such flows. One of the significant reason is that handling the subsequent complex expressions is very problematic. However, these simultaneous ramped conditions have imperative utilities such as heart deceases diagnoses and working analysis of vessels of blood. Kundu proposed a cancer treatment, based on a therapy inculcating several types of boundary conditions along with ramped wall conditions, which has no side harm for human body [21]. Extensively, these conditions have vital association with human health and daily life related problems like use of Ergometer or treadmil testing for diagnoses of cardiovascular deceases [22]. Further, Astrand and Rodahl [23], Bruce [24], and Myers and Bellin [25] played their role to enhance the effectiveness of treadmil testing.

Initially, the idea of combined ramped boundary conditions was introduced by Ahmed and Dutta [26] to analyze the flow over an infinite vertical plate. Seth et al. [27,28,29] investigated thermal and momentum profiles with ramped temperature conditions for stretching vertical sheets. The effect of wall heating on mass and energy curves for infinite vertical plate was studied by Narahari et al. [30]. Recently, Chandran et al. [31] observed the variation in momentum boundary layer thickness subjected to ramped temperature condition. Zin et al. [32] extended the study of Khan [33] on MHD flow of Jeffery fluid for ramped wall temperature. Maqbool et al. [34] further extended this work for ramped wall velocity condition to examine the significance of simultaneous boundary conditions. Mazhar et al. [35] conducted a study to observe the mass and energy behavior for Oldroyd-B fluid subjected to simultaneous ramped conditions. More practical utilities of ramped wall conditions can be seen from the contribution of Schetz [36], Hayday [37] and Malhotra [38].

On the basis of such strong motivation, we have considered incompressible, time-dependent MHD convection flow. Moreover, heat suction/injection is also introduced to the flow with the existence of a porous medium. The ramped velocity and ramped temperature conditions are considered at the wall simultaneously. Laplace transformation is implemented to reach out to the solutions.

## 2. Mathematical Modeling and Formulation of Problem

The unsteady, incompressible and magneto-hydrodynamics motion of Oldroyd-B nanofluid over an infinite vertical plate under the Boussinesq’s approximations [39] can be governed by the the succeeding equations [40,41].

(1) $$\begin{array}{c} \nabla \cdot V=0, \end{array}$$

(2) $$\begin{array}{c} \rho_{nf}\left[\frac{\partial V}{\partial t}+\left(V.\nabla \right)V\right]=\mathrm{div}T+J\times B+g{\left(\rho \beta \right)}_{nf}\left(T-T_{\infty }\right)+r, \end{array}$$

where $\rho_{nf}$, r, B, J, g, β, T, $T_{\infty }$ and t represent nanofluid density, Darcy’s resistence, total magnetic field, current density, standard gravitational force, constant of thermal volume expansion, temperature of nanofluid, ambient temperature and time respectively. Moreover, velocity V, accounting one-dimensional and uni-directional flow and the Cauchy stress tensor T are defined as

(3) $$\begin{array}{cc} V & =[u(y,t),0,0], \end{array}$$

(4) $$\begin{array}{cc} T & =-PI+S, \end{array}$$

where S and −PI denote the extra stress tensor and indeterminate stress tensor respectively. Moreover, S holds the following relation

(5) $$\begin{array}{c} \mu_{nf}\left(1+\lambda_{r}\frac{D}{Dt}\right)A_{1}=S\left(1+\lambda \frac{D}{Dt}\right), \end{array}$$

where $\mu_{nf}$ refers to dynamic viscosity of nanofluid. $\lambda_{r}$ and λ refers to retardation and relaxation time respectively. Additionally, material time derivative $\frac{D}{Dt}$ and Rivlin-Ericksen tensor $A_{1}$ are defined as

(6) $$\begin{array}{cc} \frac{DS}{Dt} & =\frac{\partial S}{\partial t}+u\frac{\partial S}{\partial x}+v\frac{\partial S}{\partial y}+w\frac{\partial S}{\partial z}, \end{array}$$

(7) $$\begin{array}{cc} A_{1} & =\nabla V+{\left(\nabla V\right)}^{T}=\left(\begin{array}{cc} 0 & u_{y}\\ u_{y} & 0 \end{array}\right). \end{array}$$

For Oldroyd-B nanofluid, modified Darcy’s law is defined as

(8) $$\begin{array}{c} -\frac{\mu_{nf}\phi }{k}\left(1+\lambda_{r}\frac{\partial }{\partial t}\right)V=\left(1+\lambda \frac{\partial }{\partial t}\right)r, \end{array}$$

where k and ϕ denote permeability and porosity of the medium respectively. The equations of Maxwell are given as

(9) $$\begin{array}{c} \mathrm{div}B=0,\;\mathrm{curl}B=\mu_{m}J,\;\mathrm{curl}E=-\frac{\partial B}{\partial t}, \end{array}$$

and

(10) $$\begin{array}{c} J\times B=-(\sigma_{\mathrm{nf}}B_{0}^{2}u,0,0), \end{array}$$

where $\mu_{m}$, $\sigma_{nf}$ and E refer to magnetic permeability, electrical conductivity of nanofluid and electric field respectively. The total magnetic field is given as $B=B_{0}+b_{0}$. Here, $B_{0}$ denotes the magnetic field applied and $b_{0}$ denotes the magnetic field induced.

In the presence of Equations (3)–(7), simplified form of (2) can be presented as

(11) $$\begin{array}{c} \rho_{nf}\frac{\partial u}{\partial t}={\left(\rho \beta \right)}_{nf}g\left(T-T_{\infty }\right)+r_{x}+{\left(J\times B\right)}_{x}+\frac{\partial S_{xy}}{\partial y}. \end{array}$$

On using Maxwell’s equations and modified Darcy’s law in above equation and multiplying it by $\left(1+\lambda \partial_{t}\right)$, we obtain the following form

$$\begin{array}{cc} \left(1+\lambda \partial_{t}\right)\rho_{nf}\frac{\partial u}{\partial t} & =\left(1+\lambda \partial_{t}\right){\left(\rho \beta \right)}_{nf}g\left(T-T_{\infty }\right)-\left(1+\lambda_{r}\partial_{t}\right)\frac{\mu_{nf}\phi }{k}u \end{array}$$

(12) $$\begin{array}{c} -\left(1+\lambda \partial_{t}\right)\sigma_{nf}B_{0}^{2}u+\left(1+\lambda \partial_{t}\right)\frac{\partial S_{xy}}{\partial y}. \end{array}$$

Plugging relation $\left(1+\lambda \partial_{t}\right)S_{xy}=\mu_{nf}\left(1+\lambda_{r}\partial t\right)u_{y}$ into the above equation and rearranging the resulted equation leads to form mentioned below

$$\begin{array}{cc} \rho_{nf}\left(1+\lambda \partial_{t}\right)\frac{\partial u}{\partial t} & =\mu_{nf}\left(1+\lambda_{r}\partial_{t}\right)\frac{\partial^{2}u}{\partial y^{2}}+g{\left(\rho \beta \right)}_{nf}\left(1+\lambda \partial_{t}\right)\left(T-T_{\infty }\right) \end{array}$$

(13) $$\begin{array}{c} -\sigma_{nf}B_{0}^{2}\left(1+\lambda \partial_{t}\right)u-\frac{\mu_{nf}\phi }{k}\left(1+\lambda_{r}\partial_{t}\right)u. \end{array}$$

**Figure 3:**
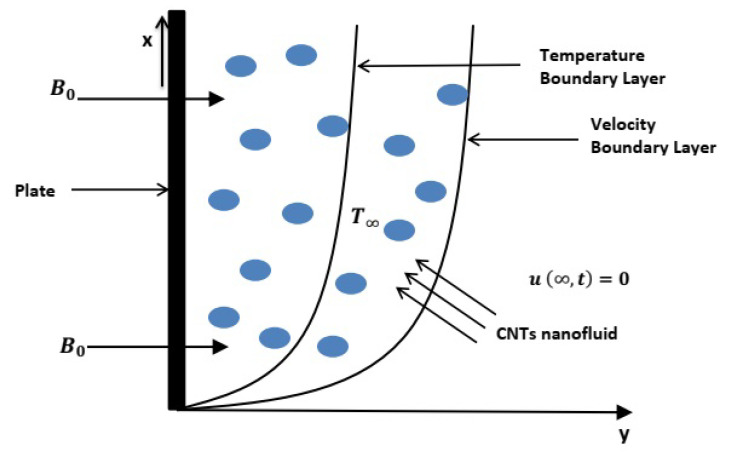
Geometrical presentation of flow.

The geometrical presentation of considered model is provided in Figure 3.

The governing equations of mass, shear stress and energy transfer under Boussinesq’s approximation incorporating carbon nanotubes are provided as

$$\begin{array}{c} \left(1+\lambda \frac{\partial }{\partial t}\right)\frac{\partial u}{\partial t}=\nu_{nf}\left(1+\lambda_{r}\frac{\partial }{\partial t}\right)\frac{\partial^{2}u}{\partial y^{2}}+\frac{1}{\rho_{nf}}{\left(\rho \beta \right)}_{nf}g\left(1+\lambda \frac{\partial }{\partial t}\right)\left(T-T_{\infty }\right) \end{array}$$

(14) $$\begin{array}{c} -\frac{\sigma_{nf}B_{0}^{2}}{\rho_{nf}}\left(1+\lambda \frac{\partial }{\partial t}\right)u-\frac{\nu_{nf}\phi }{k}\left(1+\lambda_{r}\frac{\partial }{\partial t}\right)u, \end{array}$$

(15) $$\begin{array}{c} \left(1+\lambda \frac{\partial }{\partial t}\right)\tau =\mu_{nf}\left(1+\lambda_{r}\frac{\partial }{\partial t}\right)\frac{\partial u}{\partial y}, \end{array}$$

(16) $$\begin{array}{c} {\left(\rho c_{p}\right)}_{nf}\frac{\partial T}{\partial t}=k_{nf}\frac{\partial^{2}T}{\partial y^{2}}+Q_{0}\left(T-T_{\infty }\right), \end{array}$$

where $k_{nf},{\left(\rho c_{p}\right)}_{nf}$ and $Q_{0}$ denote the nanofluid thermal conductivity, nanofluid heat capacitance and heat injection/suction respectively.

The interesting initial and boundary conditions involving ramped velocity and ramped temperature conditions at wall are defined as

$$\begin{array}{cc} u(y,0) & =0,\;T\left(y,0\right)=T_{\infty }, \end{array}$$

(17) $$\begin{array}{cc} y\geq 0:\;u_{t}\left(y,0\right) & =0,\;u_{y}\left(y,0\right)=0, \end{array}$$

(18) $$\begin{array}{cc} t>0:\;u(y,t) & \to 0,\;T\left(y,t\right)\to T_{\infty },\;\mathrm{for}\;y\to \infty ,\\ u(0,t) & =\left\{\begin{array}{c} u_{c}\frac{t}{t_{0}}\;0<t\leq t_{0}\\ u_{c}\;\;t>t_{0}, \end{array}\right. \end{array}$$

(19) $$\begin{array}{cc} T(0,t) & =\left\{\begin{array}{c} T_{\infty }+\left(T_{w}-T_{\infty }\right)\frac{t}{t_{0}}\;0<t\leq t_{0}\\ T_{w}\;\;\;\;t>t_{0}. \end{array}\right. \end{array}$$

The expressions for viscosity $\mu_{nf}$, heat capacity ${\left(c_{p}\right)}_{nf}$, coefficient of thermal expansion $\beta_{nf}$, density $\rho_{nf}$ and electrical conductivity $\sigma_{nf}$ are given as [42,43]

$$\begin{array}{c} \mu_{nf}=\frac{\mu_{f}}{{\left(1-\phi \right)}^{2.5}},\;{\left(\rho c_{p}\right)}_{nf}={\left(\rho c_{p}\right)}_{f}\left[\left(1-\phi \right)+\phi \frac{{\left(\rho c_{p}\right)}_{cnt}}{{\left(\rho c_{p}\right)}_{f}}\right],\\ {\left(\rho \beta \right)}_{nf}={\left(\rho \beta \right)}_{f}\left[\left(1-\phi \right)+\phi \frac{{\left(\rho \beta \right)}_{cnt}}{{\left(\rho \beta \right)}_{f}}\right],\;\rho_{nf}=\rho_{f}\left[\left(1-\phi \right)+\phi \frac{\rho_{cnt}}{\rho_{f}}\right],\\ \sigma_{nf}=\sigma_{f}+\frac{3\sigma_{f}\left(\sigma -1\right)\phi }{(\sigma +2)-(\sigma -1)\phi },\;\sigma =\frac{\sigma_{cnt}}{\sigma_{f}}. \end{array}$$

For thermal conductivity of CNTs, we have chosen Xue’s model [44], because it incorporates the effect of space distribution on CNTs and also embrace the rotational elliptical nanotubes with huge axial ratios.

$$\begin{array}{c} \frac{k_{nf}}{k_{f}}=\frac{1+2\phi \left(\frac{k_{cnt}}{k_{cnt}-k_{f}}\right)ln\left(\frac{k_{cnt}+k_{f}}{2k_{f}}\right)-\phi }{1+2\phi \left(\frac{k_{f}}{k_{cnt}-k_{f}}\right)ln\left(\frac{k_{cnt}+k_{f}}{2k_{f}}\right)-\phi }. \end{array}$$

On using non-dimensional terms given below [34]

(20) $$\begin{array}{c} u^{*}=\frac{u}{u_{c}},\;\xi =\frac{yu_{c}}{\nu },\;\tau^{*}=\frac{\nu \tau }{\mu u_{c}^{2}},\;t_{0}=\frac{\nu }{u_{c}^{2}},\;t^{*}=\frac{tu_{c}^{2}}{\nu },\;\theta =\frac{T-T_{\infty }}{T_{w}-T_{\infty }}. \end{array}$$

in Equations (14)–(16), and for sake of brevity, eliminating * notation, we obtain

$$\begin{array}{cc} \left(1+\lambda_{1}\frac{\partial }{\partial t}\right)\frac{\partial u}{\partial t} & =\frac{\varphi_{6}}{\varphi_{3}}\left(1+\lambda_{2}\frac{\partial }{\partial t}\right)\frac{\partial^{2}u}{\partial \xi^{2}}-M\frac{\varphi_{4}}{\varphi_{3}}\left(1+\lambda_{1}\frac{\partial }{\partial t}\right)u \end{array}$$

(21) $$\begin{array}{c} -\frac{1}{K}\frac{\varphi_{6}}{\varphi_{3}}\left(1+\lambda_{2}\frac{\partial }{\partial t}\right)u+Gr\frac{\varphi_{5}}{\varphi_{3}}\left(1+\lambda_{1}\frac{\partial }{\partial t}\right)\theta , \end{array}$$

(22) $$\begin{array}{cc} \left(1+\lambda_{1}\frac{\partial }{\partial t}\right)\tau & =\varphi_{6}\left(1+\lambda_{2}\frac{\partial }{\partial t}\right)\frac{\partial u}{\partial \xi }, \end{array}$$

(23) $$\begin{array}{cc} \frac{\partial \theta }{\partial t} & =\frac{1}{Pr}\frac{\varphi_{2}}{\varphi_{1}}\frac{\partial^{2}\theta }{\partial \xi^{2}}+\frac{Q}{\varphi_{1}}\theta , \end{array}$$

where non-dimensional quantities are defined as

$$\begin{array}{c} Gr=\frac{g\beta_{f}\nu_{f}\Delta T}{u_{c}^{3}},\;M=\frac{\sigma_{f}B_{0}^{2}\nu_{f}}{\rho_{f}u_{c}^{2}},\;Pr={\left(\frac{\mu c_{p}}{k}\right)}_{f},\;\lambda_{1}=\frac{\lambda u_{c}^{2}}{\nu_{f}},\\ \lambda_{2}=\frac{\lambda_{r}u_{c}^{2}}{\nu_{f}},\;\frac{1}{K}=\frac{\phi \nu_{f}^{2}}{k_{f}u_{c}^{2}},\;Q=\frac{\nu_{f}Q_{0}}{{\left(\rho c_{p}\right)}_{f}u_{c}^{2}}. \end{array}$$

The constants used in non-dimensional equations are given as

$$\begin{array}{c} \varphi_{1}=1-\phi +\phi \frac{{\left(\rho c_{p}\right)}_{cnt}}{{\left(\rho c_{p}\right)}_{f}},\;\varphi_{2}=\frac{1+2\phi \left(\frac{k_{cnt}}{k_{cnt}-k_{f}}\right)ln\left(\frac{k_{cnt}+k_{f}}{2k_{f}}\right)-\phi }{1+2\phi \left(\frac{k_{f}}{k_{cnt}-k_{f}}\right)ln\left(\frac{k_{cnt}+k_{f}}{2k_{f}}\right)-\phi },\;\varphi_{3}=1-\phi +\phi \frac{\rho_{cnt}}{\rho_{f}},\\ \;\varphi_{4}=1+\frac{3(\sigma -1)\phi }{(\sigma +2)-(\sigma -1)\phi },\;\varphi_{5}=1-\phi +\phi \frac{{\left(\rho \beta \right)}_{cnt}}{{\left(\rho \beta \right)}_{f}},\;\varphi_{6}=\frac{1}{{\left(1-\phi \right)}^{2.5}}. \end{array}$$

The non-dimensional form of initial and boundary conditions turn out as

(24) $$\begin{array}{cc} u(\xi ,0) & =0,\;\theta (\xi ,0)=0,\\ \xi \geq 0:\;u_{t}\left(\xi ,0\right) & =0,\;u_{\xi }\left(\xi ,0\right)=0, \end{array}$$

(25) $$\begin{array}{cc} t>0:\;u(\xi ,t) & \to 0,\;\theta (\xi ,t)\to 0\;\mathrm{when}\;\xi \to \infty , \end{array}$$

(26) $$\begin{array}{cc} u(0,t) & =\theta \left(0,t\right)=\left\{\begin{array}{c} t\;0<t\leq 1\\ 1\;t>1. \end{array}\right. \end{array}$$

## 3. Analytical Solutions

To derive the analytical solutions of current problem, Laplace transform [45] is an effective tool due to its efficient utility for non uniform boundary conditions. The formulation of Laplace transform pair in integral form to evaluate the solutions of present problem is provided as

(27) $$\begin{array}{c} \bar{W}\left(\xi ,s\right)=\int_{0}^{\infty }e^{-st}W\left(\xi ,t\right)dt=L\left[W\right]\left(t\right),\;\;t\geq 0, \end{array}$$

where W∈{θ,τ,u}. The above integral is convergent for $Re\left(s\right)>\beta_{0}$, where s=Ψ+IΩ, $\beta_{0}$ is real number and $I=\sqrt{-1}$. Laplace domain solutions can be inverted back to real time domain *t* such as

(28) $$\begin{array}{c} W\left(\xi ,t\right)=\frac{1}{2\pi I}\int_{BR}e^{st}\bar{W}\left(\xi ,s\right)ds=L^{-1}\left[\bar{W}\right]\left(s\right). \end{array}$$

### 3.1. Temperature Field

Implementation of Laplace transform on Equations $\left(23\right),{\left(25\right)}_{2}$ and ${\left(26\right)}_{2}$, and using initial condition yields

(29) $$\begin{array}{c} \frac{1}{Pr}\frac{\varphi_{2}}{\varphi_{1}}\frac{d^{2}\bar{\theta }}{d\xi^{2}}+\left(\frac{Q}{\varphi_{1}}-s\right)\bar{\theta }=0, \end{array}$$

(30) $$\begin{array}{c} \bar{\theta }\left(0,s\right)=\frac{1-e^{-s}}{s^{2}},\;\bar{\theta }\left(\xi ,s\right)\to 0\;\mathrm{as}\;\xi \to \infty . \end{array}$$

The solution of ordinary differential Equation (29) under conditions in Equation (30) is obtained as

(31) $$\begin{array}{c} \bar{\theta }\left(\xi ,s\right)=\left(\frac{1-e^{-s}}{s^{2}}\right)e^{-\sqrt{\alpha (s-a_{2})}\xi }, \end{array}$$

where

$$\begin{array}{c} \alpha =\frac{Pr\varphi_{1}}{\varphi_{2}},\;a_{2}=\frac{Q}{\varphi_{1}}. \end{array}$$

### 3.2. Velocity Field

Applying Laplace transform on Equations $\left(21\right),{\left(25\right)}_{1}$ and ${\left(26\right)}_{1}$, and using initial condition emits

$$\begin{array}{c} \frac{\varphi_{6}}{\varphi_{3}}\left(1+\lambda_{2}s\right)\frac{d^{2}\bar{u}}{d\xi^{2}}- \end{array}$$

(32) $$\begin{array}{c} \left[\left(1+\lambda_{1}s\right)s+M\frac{\varphi_{4}}{\varphi_{3}}\left(1+\lambda_{1}s\right)+\frac{1}{K}\frac{\varphi_{6}}{\varphi_{3}}\left(1+\lambda_{2}s\right)\right]\bar{u}=-Gr\frac{\varphi_{5}}{\varphi_{3}}\left(1+\lambda_{1}s\right)\bar{\theta }, \end{array}$$

(33) $$\begin{array}{c} \bar{u}\left(0,s\right)=\frac{1-e^{-s}}{s^{2}},\;\bar{u}\left(\xi ,s\right)\to 0\;\mathrm{as}\;\xi \to \infty . \end{array}$$

Plugging Equation (31) into Equation (32) and simplifying yields

(34) $$\begin{array}{c} \frac{d^{2}\bar{u}}{d\xi^{2}}-\left[\frac{\lambda_{1}s^{2}+s\left(1+\lambda_{1}a_{4}+\lambda_{2}a_{5}\right)+\left(a_{4}+a_{5}\right)}{a_{3}\left(1+\lambda_{2}s\right)}\right]\bar{u}=\\ -\frac{a_{6}}{a_{3}}\left(\frac{1+\lambda_{1}s}{1+\lambda_{2}s}\right)\left(\frac{1-e^{-s}}{s^{2}}e^{-\sqrt{\alpha (s-a_{2})}\xi }\right), \end{array}$$

where

$$\begin{array}{c} a_{3}=\frac{\varphi_{6}}{\varphi_{3}},\;a_{4}=M\frac{\varphi_{4}}{\varphi_{3}},\;a_{5}=\frac{1}{K}\frac{\varphi_{6}}{\varphi_{3}},\;a_{6}=Gr\frac{\varphi_{5}}{\varphi_{3}}. \end{array}$$

The solution of Equation (34) under conditions in Equation (33) is simplified as

(35) $$\begin{array}{c} \bar{u}\left(\xi ,s\right)=\bar{G}\left(\xi ,s\right)\left(\frac{1-e^{-s}}{s^{2}}\right), \end{array}$$

where

(36) $$\begin{array}{c} \bar{G}\left(\xi ,s\right)=e^{-\sqrt{\gamma }\xi }+\frac{a_{6}\left(1+\lambda_{1}s\right)}{\left(\alpha a_{3}\lambda_{2}-\lambda_{1}\right)\left[{\left(s-b_{1}\right)}^{2}-b_{2}^{2}\right]}\left(e^{-\sqrt{\gamma }\xi }-e^{-\sqrt{\alpha (s-a_{2})}\xi }\right), \end{array}$$

with

(37) $$\begin{array}{c} \gamma =\frac{\lambda_{1}s^{2}+s\left(1+\lambda_{1}a_{4}+\lambda_{2}a_{5}\right)+\left(a_{4}+a_{5}\right)}{a_{3}\left(1+\lambda_{2}s\right)},\;b_{1}=\frac{1-\alpha a_{3}+\lambda_{1}a_{4}+\lambda_{2}\left(a_{5}+\alpha a_{2}a_{3}\right)}{2(\alpha a_{3}\lambda_{2}-\lambda_{1})},\\ b_{2}=\sqrt{{\left(\frac{1-\alpha a_{3}+\lambda_{1}a_{4}+\lambda_{2}\left(a_{5}+\alpha a_{2}a_{3}\right)}{2(\alpha a_{3}\lambda_{2}-\lambda_{1})}\right)}^{2}+\frac{a_{4}+a_{5}+\alpha a_{2}a_{3}}{\alpha a_{3}\lambda_{2}-\lambda_{1}}}. \end{array}$$

### 3.3. Shear Field

Taking Laplace transform of Equation (22) gives

(38) $$\begin{array}{c} \left(1+\lambda_{1}s\right)\bar{\tau }=\varphi_{6}\left(1+\lambda_{2}s\right)\frac{d\bar{u}}{d\xi }. \end{array}$$

On differentiating Equation (35) with respect to variable ξ, we obtain

(39) $$\begin{array}{c} \frac{d\bar{u}}{d\xi }=\bar{H}\left(\xi ,s\right)\left(\frac{1-e^{-s}}{s^{2}}\right), \end{array}$$

where

$$\begin{array}{c} \bar{H}\left(\xi ,s\right)=-\sqrt{\gamma }e^{-\sqrt{\gamma }\xi }-\frac{a_{6}\left(1+\lambda_{1}s\right)\sqrt{\gamma }e^{-\sqrt{\gamma }\xi }}{\left(\alpha a_{3}\lambda_{2}-\lambda_{1}\right)\left[{\left(s-b_{1}\right)}^{2}-b_{2}^{2}\right]}+\\ \frac{a_{6}\left(1+\lambda_{1}s\right)\sqrt{\alpha (s-a_{2})}e^{-\sqrt{\alpha (s-a_{2})}\xi }}{\left(\alpha a_{3}\lambda_{2}-\lambda_{1}\right)\left[{\left(s-b_{1}\right)}^{2}-b_{2}^{2}\right]}. \end{array}$$

Plugging Equation (39) in Equation (38), we get

(40) $$\begin{array}{c} \bar{\tau }\left(\xi ,s\right)=\frac{\varphi_{6}\left(1+\lambda_{2}s\right)}{1+\lambda_{1}s}\left(\frac{1-e^{-s}}{s^{2}}\right)\bar{H}\left(\xi ,s\right). \end{array}$$

Since the Laplace domain solutions of temperature, momentum and shear stress in Equations (31),(35) and (40) are the multivalued functions of the Laplace parameter “*s*”, therefore numerical inversion named the Durbin method [46] is used to transform back the solution in real time domain *t*.

### 3.4. Nusselt Number

The expression for Nusselt number Nu is

(41) $$\begin{array}{cc} Nu & =-\frac{\partial \theta }{\partial \xi }{|}_{\xi =0}, \end{array}$$

(42) $$\begin{array}{cc} Nu & =L^{-1}\left[\sqrt{\alpha (s-a_{2})}\left(\frac{1-e^{-s}}{s^{2}}\right)\right]. \end{array}$$

## 4. Special Cases

This section deals with two special cases of current work.

### 4.1. Case 1

The energy and mass solutions of regular Oldroyd-B fluid (ϕ=0) with simultaneous ramped wall conditions can be deduced as:

(43) $$\begin{array}{c} \theta \left(\xi ,t\right)=L^{-1}\left[\left(\frac{1-e^{-s}}{s^{2}}\right)e^{-\sqrt{Pr(s-Q)}\xi }\right], \end{array}$$

(44) $$\begin{array}{c} u\left(\xi ,t\right)=L^{-1}\left[\left(\frac{1-e^{-s}}{s^{2}}\right)\bar{G}\left(\xi ,s\right)\right], \end{array}$$

where

$$\begin{array}{c} \bar{G}\left(\xi ,s\right)=e^{-\sqrt{\frac{a_{1}^{*}+s^{2}\lambda_{1}+a_{2}^{*}}{\lambda_{2}s+1}}\xi }+\frac{Gr(1+\lambda_{1}s)}{\left(\lambda_{2}Pr-\lambda_{1}\right)\left({\left(s-z_{1}\right)}^{2}-z_{2}^{2}\right)}\left[e^{-\sqrt{\frac{a_{1}^{*}+s^{2}\lambda_{1}+a_{2}^{*}}{\lambda_{2}s+1}}\xi }-e^{-\sqrt{Pr(s-Q)}\xi }\right], \end{array}$$

with

$$\begin{array}{c} z_{1}=\frac{a_{2}^{*}+\lambda_{2}PrQ-Pr}{2(\lambda_{2}Pr-\lambda_{1})},\;z_{2}=\sqrt{{\left(\frac{a_{2}^{*}+\lambda_{2}PrQ-Pr}{2(\lambda_{2}Pr-\lambda_{1})}\right)}^{2}+\frac{a_{1}^{*}+PrQ}{\lambda_{2}Pr-\lambda_{1}}},\;\;\\ a_{1}^{*}=M+\frac{1}{K},\;a_{2}^{*}=1+\lambda_{1}M+\frac{\lambda_{2}}{K}. \end{array}$$

### 4.2. Case 2

The energy and mass results of Oldroyd-B nanofluid for constant boundary conditions can be obtained as:

(45) $$\begin{array}{c} \theta \left(\xi ,t\right)=e^{-\xi \dot{ı}\sqrt{\alpha a_{2}}}\mathrm{erfc}\left(\frac{\xi \sqrt{a_{2}}}{2\sqrt{t}}-\dot{ı}\sqrt{a_{2}t}\right)+e^{\xi \dot{ı}\sqrt{\alpha a_{2}}}\mathrm{erfc}\left(\frac{\xi \sqrt{a_{2}}}{2\sqrt{t}}+\dot{ı}\sqrt{a_{2}t}\right), \end{array}$$

(46) $$\begin{array}{c} u\left(\xi ,t\right)=L^{-1}\left[\left(\frac{1-e^{-s}}{s}\right)\bar{G}\left(\xi ,s\right)\right], \end{array}$$

where

$$\begin{array}{c} \bar{G}\left(\xi ,s\right)=e^{-\sqrt{\gamma }\xi }+\frac{a_{6}\left(1+\lambda_{1}s\right)}{\left(\alpha a_{3}\lambda_{2}-\lambda_{1}\right)\left[{\left(s-b_{1}\right)}^{2}-b_{2}^{2}\right]}\left(e^{-\sqrt{\gamma }\xi }-e^{-\sqrt{\alpha (s-a_{2})}\xi }\right), \end{array}$$

with γ, $b_{1}$ and $b_{2}$ provided in Equation (37).

The purpose of comprehensive understanding of the physics of the current problem is served with the help of parametric study and variation in solutions is elucidated with the support of tables and graphs. The solutions presented in these graphs are of four kinds. Plot (a) and (b) in every figure present the results for SWCNTs and MWCNTs respectively. The results for isothermal plate and ramped plate are represented by dashed lines and solid lines respectively. Thermophysical properties of base fluid and two types of CNTs, i.e., SWCNTs and MWCNTs, are provided in Table 1. In order to find the effective values of thermal conductivity of CNTs for several values of volume fraction ϕ, model proposed by Xue [44] is utilized and a comparison between thermal conductivity for SWCNTs and MWCNTs is provided in Table 2. It is spotted that for the same values of volume fraction, nanofluid with MWCNTs have lower thermal conductivity in contrast to nanofluid with SWCNTs. This is physically justified by the fact that MWCNTs have lower thermal conductivity which is 3000 W/mK in contrast to thermal conductivity of SWCNTs which is 6600 W/mK. In Table 3, alteration in Nusselt number under variation of different parameters for SWCNTs and MWCNTs is given to understand the effects of those parameters on heat transfer. From Table 3, enhancement in heat transfer is pretty clear with maximization of volume fraction of carbon nanotubes. This kind of behavior was expected to show the significance of nanofluids in practical purposes such as heating and cooling processes. It can be stated as well that for each parameter, rate of heat transfer for SWCNTs is slightly higher than that of MWCNTs. This fact also justifies the little difference in the heights of graphs of SWCNTs and MWCNTs in the case of each associated parameter.

In order have deep insight into the relative difference between temperature and velocity profiles of sodium alginate based nanofluid having SWCNTs and MWCNTs as nanoparticles, both solutions are tabulated in Table 4 and Table 5. The corresponding tables describe that in case of ϕ=0 (i.e., pure Sodium alginate) we have the same values of SWCNTs and MWCNTs mass and energy solutions. It can be seen from Table 4 that temperature has higher profile in case of SWCNTs because of their relatively higher thermal conductivity. This factor also points out the little difference in heights of solutions for SWCNTs and MWCNTs. Table 4 shows that temperature is an increasing function of ϕ,t and Q>0 and decreasing function of Q<0 for both SWCNTs and MWCNTs. Table 5 provides that solution of velocity has higher values in case of MWCNTs because of their relatively lower density. It also concludes that velocity solution faces similar kind of influence for both SWCNTs and MWCNTs. It is observed from the table that velocity is an elevating function of $\phi ,\lambda_{2},t,Gr$ and *K*, while on the other hand fluid is decelerated by increasing values of *M* and $\lambda_{1}$. Table 6 illustrates variation in wall shear stress when other associated quantities are altered. It can be concluded from the table that velocity on plate is a decreasing function of $\lambda_{1}$ and ϕ while it behaves inversely for $\lambda_{2}$ and *K*.

## 5. Results and Discussion

**Figure 4:**
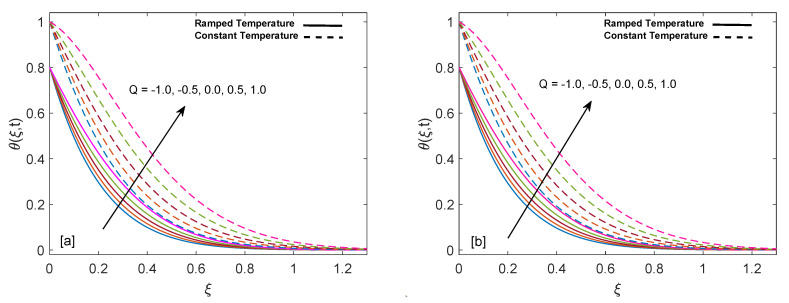
Temperature behavior for variation in Q at t=0.8 and ϕ=0.02.

**Figure 5:**
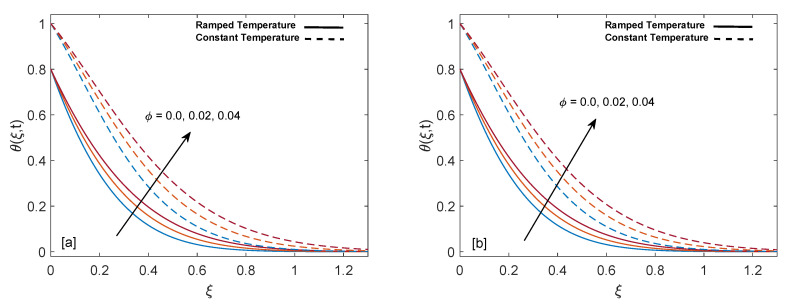
Temperature behavior for variation in ϕ at t=0.8 and Q=0.5.

**Figure 6:**
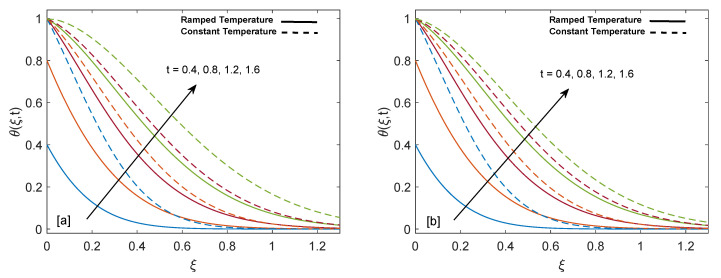
Temperature behavior for variation in *t* at Q=0.5 and ϕ=0.02.

Significance of heat suction/injection parameter (Q) in rise or fall of temperature is graphed in Figure 4a,b. Positive values of Q are referred to heat injection and negative values of Q are associated with heat suction. The graph describes that increase in positive value of Q rises the temperature but on the other hand increase in negative value of Q drops the temperature. Physically, increase in positive value of Q means more heat is injected, so temperature must increase, as shown in the graph. Likewise, increase in negative value of Q corresponds to more suction or consumption of heat, which means that temperature must decrease. Moreover, in case of constant wall temperature, solution has higher profile as compared to ramped wall temperature. Figure 5a,b display alteration in temperature values due to variation in volume fraction (ϕ) of nanoparticles. As ϕ enlarges, temperature boundary layer thickness increases, which is justified by the physical behavior of nanoparticles. Moreover, the temperature boundary layer is greater for sodium alginate based nanofluid as compared to pure sodium alginate (ϕ=0). The reason is higher thermal conductivity of CNTs, which consequently raises the thermal conductivity of base fluid when CNTs are added to it. Eventually, we observe elevation in temperature boundary layer thickness. This observation concludes the significance of nanoparticles in heating and cooling processes. Additionally, thermal boundary layer thickness of ramped wall temperature is less than thermal boundary layer thickness of constant wall temperature. It is presented in Figure 6a,b that as time (*t*) duration increases, temperature of fluid rises for both ramped wall and isothermal wall conditions.

**Figure 7:**
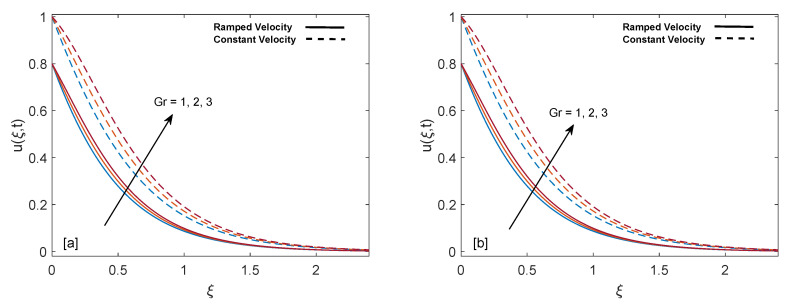
Velocity behavior for variation in Gr at $t=0.8,\lambda_{1}=1,\phi =0.02,M=2,K=0.6$ and $\lambda_{2}=1$.

Figure 7a,b describe the effect of Grashof number (Gr) on mass distribution. The thickness of momentum boundary layer in case of isothermal temperature condition is higher as compared to ramped wall temperature. It is observed that maximization of Gr elevates the mass profile. The physical logic behind this behavior is reduction of resistance. Since Gr is the fraction of buoyancy and viscous forces, increase in Gr leads to strong buoyancy force near the plate, which suppress the resistances and fluid flows more rapidly. Moreover, away from the plate the buoyancy force gets weaker and leads to calmness of fluid.

**Figure 8:**
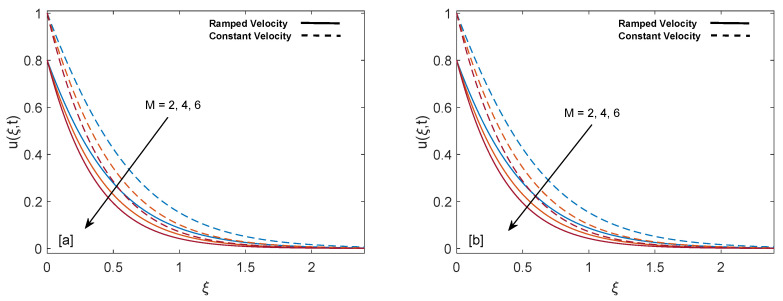
Velocity behavior for variation in M at $t=0.8,\lambda_{1}=1,\phi =0.02,Gr=1,K=0.6$ and $\lambda_{2}=1$.

Effect of magnetic parameter (M) on ramped wall velocity and constant wall velocity is illustrated in Figure 8a,b. It is witnessed that velocity of constant wall temperature is greater than that of ramped wall temperature. Velocity of fluid drops for increasing values of M because applied magnetic field leads to existence of strong Lorentz force. This force acts as a dragging force and presents strong resistance to flow of fluid, therefore eventually mass profile decreases. As fluid moves away from the plate this Lorentz force gets weaker and fluid comes to rest.

**Figure 9:**
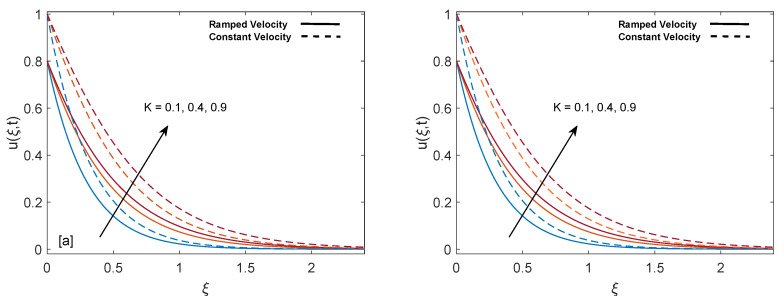
Velocity behavior for variation in K at $t=0.8,\lambda_{1}=1,\phi =0.02,M=2,Gr=1$ and $\lambda_{2}=1$.

Figure 9a,b depict the impact of porosity parameter (K) on mass profile. It is observed that mass profile gets elevation for enlargement in values of K. The physically supporting factor is reduction of friction in porous medium. When K increases, fluid faces less resistance which in turn increase the momentum development of the regime and as a result velocity profile is raised. Furthermore, velocity is low in case of ramped boundary condition.

**Figure 10:**
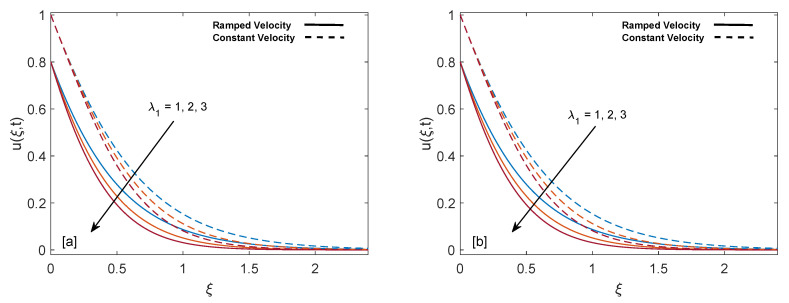
Velocity behavior for variation in $\lambda_{1}$ at t=0.8,Gr=1,ϕ=0.02,M=2,K=0.6 and $\lambda_{2}=1$.

Figure 10a,b describe the behavior of mass distribution for different values of relaxation time ($\lambda_{1}$). Momentum boundary layer thickness has greater values for isothermal wall condition as compared to ramped wall condition. As value of $\lambda_{1}$ enlarges, mass profile of fluid declines. Physically, as $\lambda_{1}$ increment implies that fluid will take extra time to get calm, it readily justifies the fall in velocity curves.

**Figure 11:**
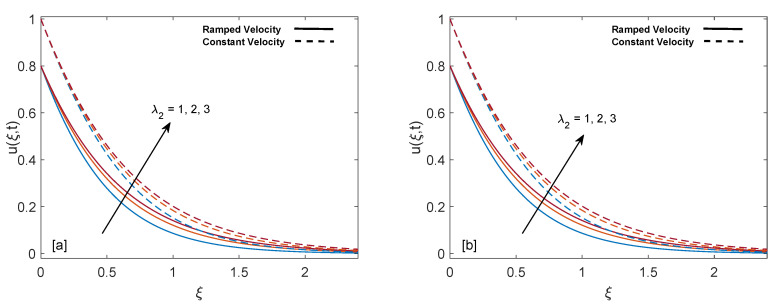
Velocity behavior for variation in $\lambda_{2}$ at $t=0.8,\lambda_{1}=1,\phi =0.02,M=2,K=0.6$ and Gr=1.

The contribution of retardation time ($\lambda_{2}$) in fluid flow is sketched in Figure 11a,b. It is noticed that momentum boundary layer thickness increases in both cases since an increase in $\lambda_{2}$ reduces the resistance. As a consequence, fluid is accelerated. Velocity for constant wall condition is greater as compared to ramped wall condition.

**Figure 12:**
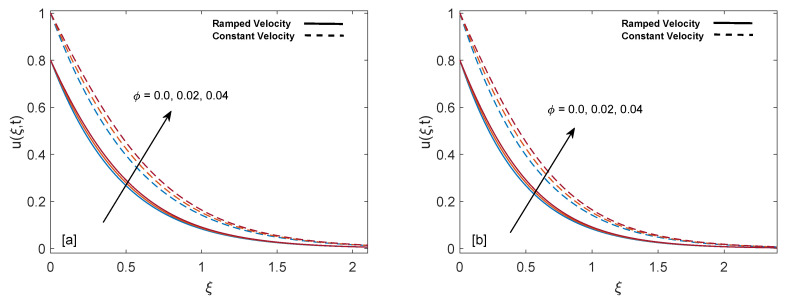
Velocity behavior for variation in ϕ at $t=0.8,\lambda_{1}=1,M=2,Gr=1,K=0.6$ and $\lambda_{2}=1$.

Figure 12a,b analyze the significance of addition of CNTs to our base fluid. It is visible from the maps that velocity is an increasing function of volume fraction for both ramped wall and constant wall conditions. This happens because suspension of CNTs in base fluid reduces the viscous forces and leads to elevation of momentum boundary layer.

**Figure 13:**
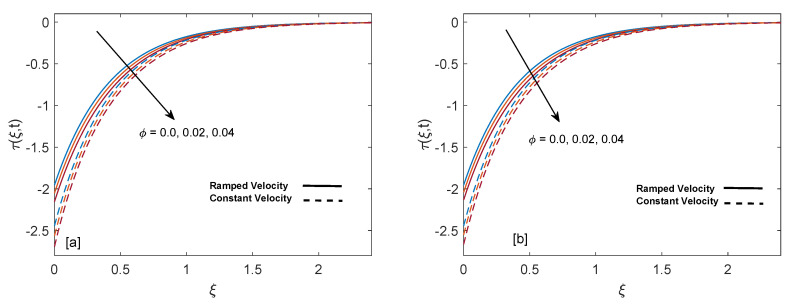
Shear stress behavior for variation in ϕ at $t=0.8,\lambda_{1}=1,M=2,Gr=1,K=0.6$ and $\lambda_{2}=1$.

**Figure 14:**
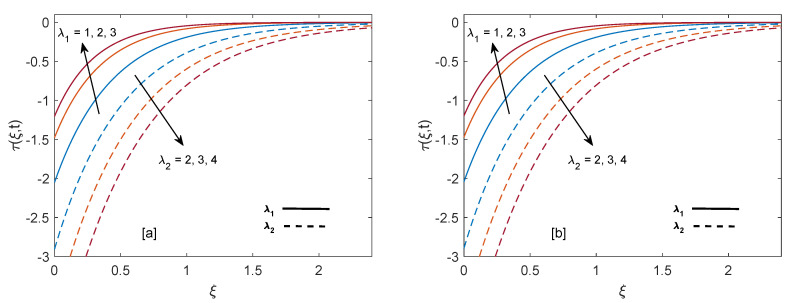
Shear stress behavior for variation in $\lambda_{1}$ and $\lambda_{2}$ at t=0.8,M=2,ϕ=0.02,Gr=1 and K=0.6.

**Figure 15:**
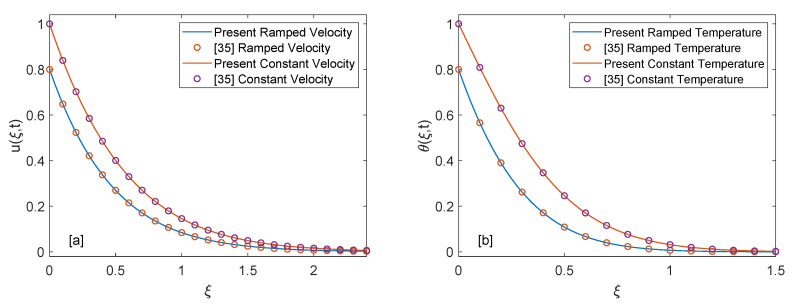
Validation of present velocity and temperature solutions.

Figure 13a,b show the shear stress curves for SWCNTs and MWCNTs for distinct values of volume fraction. It is detected that shear stress decreases with elevation of ϕ. Shear stress profiles incorporating $\lambda_{1}$ and $\lambda_{2}$ are drawn in Figure 14a,b. It is clear from the profiles that shear stress has inverse behavior for $\lambda_{1}$ and $\lambda_{2}$. As $\lambda_{1}$ increases, magnitude of shear shear stress elevates and ultimately magnitude of velocity reduces. Contrarily, increase in $\lambda_{2}$ results in enhancement of velocity due to decrease in skin friction. In order to authenticate our current solutions, Figure 15a,b are presented. It can be observed that if heat injection/suction and volume fraction of nanoparticles are removed from the current model (later case shows that only regular fluid is considered), then present solutions of velocity and temperature field are in excellent agreement with velocity and temperature solutions of [35]. This comparison verifies the present study.

## 6. Conclusions

This work aims to investigate the heat transfer enhancement when nanoparticles in the form of carbon nanotubes are suspended in a base fluid along with ramped wall velocity and ramped temperature conditions in a porous medium. It is significant to mention that the use of ramped conditions simultaneously is physically effective, but restricted in the literature, especially for nanofluids. The principal governing equations of momentum, shear stress and energy for MHD convective unsteady flow of Oldroyd-B nanofluid, are comprised of partial differential equations. These equations are solved via Laplace transform and Durbin method. The solutions of ramped wall condition are compared with those of constant wall condition. Moreover, Nusselt number expression is derived for in depth analysis of enhancement in heat transfer. The variation in solution profiles, resulting due to an increase or decrease in particular parameters, is observed with the help of graphs and tables. The solutions for single wall carbon nanotubes (SWCNTs) and multi-wall carbon nanotubes (MWCNTs) are also compared.

The significant results of this study are

- Mass profile gets elevation with increase in ϕ, Gr, K, and $\lambda_{2}$. Oppositely, an increase in relaxation time $\lambda_{1}$ and magnetic parameter M decelerate the flow.
- An increase in the amount of heat injection and volume fraction of nanoparticles enhances the temperature, while an inverse behavior is witnessed for the increase in the amount of heat suction.
- Heat transfer enhances when the volume fraction ϕ of CNTs increases. The values of ϕ are calculated using model proposed by Xue [44]. It is found that maximization in volume fraction boosts the thermal conductivity, which results in a higher rate of heat transfer.
- Velocity on the plate (skin friction) increases with an increase in retardation time $\lambda_{2}$ and behaves oppositely for relaxation time $\lambda_{1}$ and volume fraction ϕ.

## Figures and Tables

**Table 1:** Thermophysical properties of base fluid and CNTs [47,48].

Physical Properties	Sodium Alginate	SWCNTs	MWCNTs
k (W/m K)	0.613	6600	3000
ρ $\left(kg/m^{3}\right)$	989	2600	1600
$c_{p}$ (J/kg K)	4175	425	796
$\beta \times 10^{-5}$ (1/K)	0.99	27	44

**Table 2:** Variation in thermal conductivity of nanofluid for several values of volume fraction.

**Volume Fraction** (ϕ)	0.00	0.01	0.02	0.03	0.04
**Thermal Conductivity for SWCNT $\left(k_{\mathrm{nf}}\right)$**	0.145	0.174	0.204	0.235	0.266
**Thermal Conductivity for MWCNT $\left(k_{\mathrm{nf}}\right)$**	0.145	0.172	0.2	0.228	0.257

**Table 3:** Variation of Nusselt number under influence of different parameters when Pr = 12.

t	Q	ϕ	Nu for SWCNT	Nu for MWCNT
**0.6**	0.5	0.02	**3.0803**	**3.0438**
**0.7**	-	-	**3.2597**	**3.2212**
**0.8**	-	-	**3.4120**	**3.3717**
0.8	**−1**	-	**4.9698**	**4.9097**
-	**−0.5**	-	**4.4898**	**4.4358**
-	**0**	-	**3.9728**	**3.9253**
-	**0.5**	-	**3.4120**	**3.3717**
-	**1**	-	**2.7988**	**2.7664**
-	0.5	**0.0**	**3.0099**	**3.0099**
-	-	**0.02**	**3.4120**	**3.3717**
-	-	**0.04**	**3.7718**	**3.6994**

**Table 4:** Variation of temperature under influence of different parameters when Pr = 12.

y	t	Q	ϕ	Temperature of SWCNTs	Temperature of MWCNTs
0.3	0.8	0.5	**0.0**	**0.2040**	**0.2040**
-	-	-	**0.02**	**0.2521**	**0.2477**
-	-	-	**0.04**	**0.2923**	**0.2851**
-	-	**−1**	0.02	**0.1741**	**0.1704**
-	-	**−0.5**	-	**0.1958**	**0.1920**
-	-	**0**	-	**0.2215**	**0.2174**
-	-	**0.5**	-	**0.2521**	**0.2477**
-	-	**1**	-	**0.2886**	**0.2840**
-	**0.6**	0.5	-	**0.1475**	**0.1444**
-	**0.7**	-	-	**0.1975**	**0.1937**
-	**0.8**	-	-	**0.2521**	**0.2477**
**0.4**	0.8	-	-	**0.1581**	**0.1541**
**0.5**	-	-	-	**0.0950**	**0.0918**
**0.6**	-	-	-	**0.0547**	**0.0523**

**Table 5:** Variation of velocity under influence of different parameters when Pr = 12 and Q = 0.5.

y	t	ϕ	M	Gr	K	$\lambda_{1}$	$\lambda_{2}$	Velocity for SWCNT	Velocity for MWCNT
**0.3**	0.8	0.02	2.0	1.0	0.6	1.0	1.0	**0.4336**	**0.4350**
**0.4**	-	-	-	-	-	-	-	**0.3484**	**0.3499**
**0.5**	-	-	-	-	-	-	-	**0.2785**	**0.2800**
0.3	**0.6**	-	-	-	-	-	-	**0.3098**	**0.3110**
-	**0.7**	-	-	-	-	-	-	**0.3713**	**0.3726**
-	**0.8**	-	-	-	-	-	-	**0.4336**	**0.4350**
-	0.8	**0.0**	-	-	-	-	-	**0.4180**	**0.4180**
-	-	**0.02**	-	-	-	-	-	**0.4336**	**0.4350**
-	-	**0.04**	-	-	-	-	-	**0.4483**	**0.4511**
-	-	0.02	**2.0**	-	-	-	-	**0.4336**	**0.4350**
-	-	-	**4.0**	-	-	-	-	**0.3848**	**0.3862**
-	-	-	**6.0**	-	-	-	-	**0.3466**	**0.3480**
-	-	-	2.0	**1.0**	-	-	-	**0.4336**	**0.4350**
-	-	-	-	**2.0**	-	-	-	**0.4604**	**0.4620**
-	-	-	-	**3.0**	-	-	-	**0.4872**	**0.4889**
-	-	-	-	1.0	**0.1**	-	-	**0.4336**	**0.4350**
-	-	-	-	-	**0.4**	-	-	**0.4604**	**0.4620**
-	-	-	-	-	**0.9**	-	-	**0.4872**	**0.4889**
-	-	-	-	-	0.6	**1.0**	-	**0.4336**	**0.4350**
-	-	-	-	-	-	**2.0**	-	**0.3924**	**0.3943**
-	-	-	-	-	-	**3.0**	-	**0.3601**	**0.3622**
-	-	-	-	-	-	1.0	**1.0**	**0.4336**	**0.4350**
-	-	-	-	-	-	-	**2.0**	**0.4662**	**0.4673**
-	-	-	-	-	-	-	**3.0**	**0.4837**	**0.4845**

**Table 6:** Variation of wall shear stress under influence of different parameters when Pr = 12 and Q = 0.5.

t	ϕ	M	K	$\lambda_{1}$	$\lambda_{2}$	Shear Stress for SWCNT	Shear Stress for MWCNT
**0.6**	0.02	2.0	0.6	1.0	1.0	**−1.5388**	**−1.5308**
**0.7**	-	-	-	-	-	**−1.7952**	**−1.7859**
**0.8**	-	-	-	-	-	**−2.0517**	**−2.0411**
0.8	**0.0**	-	-	-	-	**−1.9546**	**−1.9546**
-	**0.02**	-	-	-	-	**−2.0517**	**−2.0411**
-	**0.04**	-	-	-	-	**−2.1544**	**−2.1329**
-	0.02	**2.0**	-	-	-	**−2.0517**	**−2.0411**
-	-	**4.0**	-	-	-	**−2.3745**	**−2.3628**
-	-	**6.0**	-	-	-	**−2.6583**	**−2.6457**
-	-	2.0	**0.1**	-	-	**−3.1796**	**−3.1727**
-	-	-	**0.4**	-	-	**−2.1907**	**−2.1808**
-	-	-	**0.9**	-	-	**−1.9535**	**−1.9423**
-	-	-	0.6	**1.0**	-	**−2.0517**	**−2.0411**
-	-	-	-	**2.0**	-	**−1.4781**	**−1.4695**
-	-	-	-	**3.0**	-	**−1.2047**	**−1.1972**
-	-	-	-	1.0	**2.0**	**−2.9071**	**−2.8946**
-	-	-	-	-	**3.0**	**−3.7164**	**−2.8946**
-	-	-	-	-	**4.0**	**−4.5045**	**−4.4899**
